# Reply to Pessoa, P.; Arderucio Costa, B. Comment on “Tsallis, C. Black Hole Entropy: A Closer Look. *Entropy* 2020, *22*, 17”

**DOI:** 10.3390/e23050630

**Published:** 2021-05-19

**Authors:** Constantino Tsallis

**Affiliations:** 1 Centro Brasileiro de Pesquisas Físicas and National Institute of Science and Technology of Complex Systems, Rua Xavier Sigaud 150, Rio de Janeiro 22290-180, Brazil; tsallis@cbpf.br; 2 Santa Fe Institute, 1399 Hyde Park Road, Santa Fe, NM 87501, USA; 3 Complexity Science Hub Vienna, Josefstädter Strasse 39, 1080 Vienna, Austria

**Keywords:** black holes, nonadditive entropies, thermodynamics, complex systems

## Abstract

In the present Reply we restrict our focus only onto the main erroneous claims by Pessoa and Costa in their recent Comment (*Entropy* **2020**, *22*, 1110).

## 1. Relevant Misunderstanding

A severe misunderstanding is present already in the first sentence of the Abstract of the Comment [1]. This also emerges in the Introduction and elsewhere. More precisely, we read in the Abstract: *“In a recent paper (Entropy* **2020***, 22, 17), Tsallis states that entropy—as in Shannon or Kullback–Leiber’s definitions—is inadequate to interpret black hole entropy and suggests that a new non-additive functional should take the role of entropy.”*

Quite regretfully, the authors paid no attention at all to a most relevant *if*. Indeed, as emphasized in [2], and explicitly written in [3], my claim is that *“In what concerns thermodynamics, the spatial dimensionality of a (3+1) black hole depends on whether its bulk (inside its event horizon or boundary) has or not non negligible amount of matter or analogous physical information. If that matter or information is non negligible, the thermodynamical entropy of the black hole must scale as $L^{d}$ with d=3, where L stands for its linear size. If that matter or information is negligible, the thermodynamic entropy of the black hole must scale as $L^{d}$ with d=2”*. Neither in [2] nor in [3] is a specific position taken for black holes being d=2+1 or d=3+1 physical objects, or even (multi)fractal objects with nonintenger *d* (which, to the best of our knowledge, may not be excluded). At the present stage, it seems appropriate to analyze this nontrivial and delicate issue within the specialized realm of cosmological and black-hole physics [4,5,6,7,8,9,10,11,12,13,14,15,16,17,18,19,20,21,22,23,24,25,26,27,28,29,30,31,32,33,34,35,36,37,38,39,40,41,42,43,44,45,46,47,48]. However, it is seemingly undeniable that the sort of perplexity expressed in [49,50,51,52], and elsewhere, emerges because, *if* black holes are thought to be d=3 objects, their thermodynamical entropy should be proportional to the cube of the radius, and not to its square, as it happens with Boltzmann–Gibbs-based Bekenstein–Hawking entropy. This entropy can be shown to yield $\frac{S_{BG}}{k}=\frac{1}{4}\frac{A_{H}}{L_{P}^{2}}$, where $L_{P}\equiv \sqrt{\frac{hG}{c^{3}}}\equiv Planck\;length$. What is basically argued in [2,3] is that the dominant term of the thermodynamical entropy *S* of a *d*-dimensional black hole whose event horizon area is $A_{H}$ is expected to satisfy $\frac{S}{k}\propto {\left(\frac{\sqrt{A_{H}}}{L_{P}}\right)}^{d}$. Therefore, if d=2, we recover the Bekenstein–Hawking entropy, but, if d≠2, a non-BG entropic functional must be used for thermodynamical purposes. The functional $S_{q,\delta }$ with specific *d*-dependent values of (q,δ) emerges as a plausible candidate for such a non-BG functional.

Before ending this Section let us emphasize what we precisely mean by “thermodynamical purposes”. We focus here on two specific aspects of this issue, which play the most central role in the present Reply.

(i) Let us assume that a system can be modelled by a long-ranged-interacting many-body problem (e.g., classical models such as the *d*-dimensional α-XY ferromagnet [53,54], α-Heisenberg ferromagnet [55,56,57,58,59], α-Fermi–Pasta–Ulam model [60,61,62,63,64,65,66,67,68], α-Lennard–Jones gas [69,70,71,72]), *N* basically being the number of particles. The notation, including α in all of them, comes from the fact that a two-body attractive interaction is assumed in all of them, which asymptotically decays as $1/r^{\alpha }$ (with α≥0), *r* being the distance. In all such cases, the corresponding Gibbs free energy is given by G(N)=U(N)−TS(N)+pV(N)−μN−HM(N)−…. We now divide by $N\tilde{N}$ where $\tilde{N}\equiv \frac{N^{1-\alpha /d}-1}{1-\alpha /d}$; $\tilde{N}$ behaves, for large values of *N*, as $N^{1-\alpha /d}/\left(1-\alpha /d\right)$ for 0≤α/d<1 (long-range interactions), as 1/(α/d−1) for α/d>1 (short-range interactions), and as lnN for α/d=1(marginally-ranged interactions). We then obtain $g=u-\tilde{T}s-+\tilde{p}v-\tilde{\mu }-\tilde{H}m-\ldots$, where $g\equiv \lim_{N\to \infty }G\left(N\right)/N\tilde{N}$, $u\equiv \lim_{N\to \infty }U\left(N\right)/N\tilde{N}$, $s\equiv \lim_{N\to \infty }S\left(N\right)/N$, $v\equiv \lim_{N\to \infty }V\left(N\right)/N$, $m\equiv \lim_{N\to \infty }M\left(N\right)/N$, $\tilde{T}\equiv \lim_{N\to \infty }T/\tilde{N}$, $\tilde{p}\equiv \lim_{N\to \infty }p/\tilde{N}$, $\tilde{\mu }\equiv \lim_{N\to \infty }\mu /\tilde{N}$, $\tilde{H}\equiv \lim_{N\to \infty }H/\tilde{N}$, …. It should be very clear at this point that *all these quantities must be finite if we wish to preserve the entire Legendre structure of thermodynamics.* These specific scalings have already been checked and found to be correct, very particularly in order to have *finite* equations of states, for diverse physical systems, such as a ferrofluid-like model [73], Lennard–Jones-like fluids [69,70,71,72], magnetic systems [74,75,76,77,78,79], anomalous diffusion [80], and percolation [81,82], among others. An immediate corollary is that quantities such as entropy *S*, *d*-dimensional volume *V*, number of particles *N*, and total magnetization *M*, belong to the same thermodynamic class, and are *extensive independently from the interactions being short- or long-ranged*. This is in remarkable contrast with quantities such as *G* and *U*, which are extensive for short-ranged interactions (implicit assumption in nearly all textbooks of thermodynamics, although not necessarily emphasizing it, quite regretfully) but *super-extensive ($\propto N^{2-\alpha /d}$) for long-ranged ones*. In particular, U(N) scales as $N^{2}$ for all mean-field models (i.e., α=0), which is the reason for nearly all authors dividing by *N* the coupling constant of the many-body Hamiltonian. Consistently, quantities such as $\tilde{T}$, $\tilde{p}$, $\tilde{\mu }$, $\tilde{H}$ are intensive, ∀α/d>0.

(ii) We provide a brief reminder of the *Large Deviation Theory* (LDT). If we throw *N* (say even) independent coins, the probability of having n≠N/2 heads is given by $P\left(N;n/N<x\right)≃e^{-r_{1}\left(x\right)\;N}$, where the *rate function*$r_{1}\left(x\right)$ is the relative BG entropy per particle and satisfies $r_{1}\left(1/2\right)=0$. In other words, $r_{1}\left(x\right)N$ corresponds to the *total* entropy, which is therefore *extensive*, as is well known for this simple case. This property mirrors the fact that, within BG statistical mechanics, the thermal equilibrium probability associated with Hamiltonian $H_{N}$ is given by $p≃e^{-\beta H_{N}}=e^{-[\beta H_{N}/N]\;N}$, where we can see that $\left[\beta H_{N}/N\right]$ is an intensive quantity which plays the role of $r_{1}\left(x\right)$. For usual systems, more specifically for those for which the Central Limit Theorem legitimately applies, both expressions, $P\left(N;n/N<x\right)≃e^{-r_{1}\left(x\right)\;N}$ and $p≃e^{-\beta H_{N}}$, still apply. However, if we have relevant nonlocal features, the CLT and the LDT need to be generalized. For nonlocally correlated elements (e.g., for classical many-body systems with 0≤α/d<1) the usual CLT and the LDT are not expected to apply. It has been verified in many of such systems that the CLT Gaussian attractor is replaced by a *q*-Gaussian one with q>1 (see, for instance, Refs. [83,84] to have a first approach to such anomalies). The corresponding stationary-state distribution optimizing, under appropriate simple constraints, the nonadditive entropy $S_{q}$ becomes $p≃e_{q}^{-\beta_{q}H_{N}}=e_{q}^{-[\left(\beta_{q}\tilde{N}\right)\left(H_{N}/N\tilde{N}\right)]\;N}$, where $\left[\left(\beta_{q}\tilde{N}\right)\left(H_{N}/N\tilde{N}\right)\right]=\left(1/\tilde{T}\right)\left(H_{N}/N\tilde{N}\right)$ is *intensive*, as shown in point (i) above. For a similar probabilistic system with strongly correlated coins (within a wide class of correlations), it is allowed to expect $P\left(N;n/N<x\right)≃e_{q}^{-r_{q}\left(x\right)\;N}$ with the *q-rate function $r_{q}\left(x\right)$* being of the order of some appropriate *q*-relative entropy per particle, and satisfying $r_{q}\left(1/2\right)=0$. As we see, if this conjecture is correct, the *total* entropy corresponds to $r_{q}\left(x\right)N$ and is, once again, extensive. This conjecture has been numerically verified with high-precision calculations in at least one non-trivial example [85,86,87]; more are coming.

## 2. About Entropic Additivity and Extensivity

The authors of the Comment write next, in the Abstract: *“Here we counterargue by explaining the important distinction between the properties of extensivity and additivity; the latter is fundamental for entropy, while the former is a property of particular thermodynamical systems that is not expected for black holes.”*

I could not agree more with Pessoa and Costa about the importance of the distinction between extensivity and additivity, very particularly when entropy is focused on. But their use of the verb ”explaining” appears to differ from that of others. The distinction additivity *versus* extensivity has been addressed in very many occasions in the context of nonextensive statistical mechanics (*q*-statistics for short) and nonadditive entropies, e.g., in [88,89] (a wide Bibliography is available at [90]). As transparently defined by Penrose [91], the *additivity* of an entropic functional $S(\left\{p_{i}\right\})$ is based on the simple mathematical property $S\left(\left\{p_{i}p_{j}\right\}\right)=S\left(\left\{p_{i}\right\}\right)+S\left(\left\{p_{j}\right\}\right)$, $\forall \{\left(p_{i},p_{j}\right)\}$, i.e., S(A+B)=S(A)+S(B), *A* and *B* being probabilistically independent systems. Consequently, it is trivially verified that the Boltzmann–Gibbs–von Neumann–Shannon (noted $S_{BG}$ here) and the Renyi entropic functionals are additive, whereas all the others available in the literature are nonadditive, among them $S_{q}$, $S_{\delta }$ and $S_{q,\delta }$ [2]. At this point, it is worth stressing that the $S_{q}$ functional has been the object of *uniqueness* theorems in what concerns (i) the axiomatic formulations by Santos and by Abe [92,93], respectively, generalizing those of Shannon and of Khinchin; (ii) the Topsoe-factorizability [94] in game theory; (iii) the Amari–Ohara–Matsuzoe conformally invariant geometry [95]; (iv) the Biro–Barnafoldi–Van thermostat universal independence [96,97,98,99,100]; (v) the Enciso–Tempesta uniqueness of composable trace-form functionals [101], thus leading to the likelihood factorization required by Einstein [102].

In strong contrast with additivity, the *extensivity* of an entropy (i.e., $0<\lim_{L\to \infty }S\left(L\right)/L^{d}<\infty$, where *L* is the linear size of the *d*-dimensional system) depends not only on the specific entropic functional but also—and very much so—on the specific system that is being focused on. What Pessoa and Costa definitively appear to miss in their Comment is that entropic additivity is physically subordinated to entropic extensivity, and not the other way around. To better understand in what sense we are using the word ”subordinated” we may refer to an analogous situation, namely the Galilean additivity of velocities ($v_{13}=v_{12}+v_{23}$). This additivity is ”subordinated” to the Lorentz invariance imposed by Einstein in order to unify mechanics with Maxwell equations, which eventually generalized the Galilean additivity into the Einstein composition of velocities involving the vacuum speed of light *c* ($v_{13}=\left(v_{12}+v_{23}\right)/\left(1+v_{12}\;v_{23}/c^{2}\right)$). As is well known, the Einstein composition of velocities recovers the Galilean additivity in the limit 1/c→0, fairly similarly to how the nonadditivity of $S_{q}$ recovers the BG additivity in the limit (1−q)/k→0 (let us remind the reader that $S_{q}\left(A+B\right)/k=S_{q}\left(A\right)/k+S_{q}\left(B\right)/k+\left(1-q\right)\left[S_{q}\left(A\right)/k\right]\left[S_{q}\left(B\right)/k\right]$, hence $S_{q}\left(A+B\right)=S_{q}\left(A\right)+S_{q}\left(B\right)+\left[\left(1-q\right)/k\right]S_{q}\left(a\right)S_{q}\left(B\right)$. The loss of the Galilean additivity is then a small price to pay for making mechanics and Maxwell electromagnetism simultaneously Lorentz-invariant. In analogy, the loss of the BG entropic additivity is, whenever necessary (i.e., whenever there is strong space–time entanglement in the system), a small price to pay for satisfying thermodynamics.

No *general* physical reason is known to necessarily lead to additive entropies for thermodynamical purposes, whereas entropic extensivity is generically mandated by the Legendre structure of thermodynamics (see [2] and many other references therein). (We read in [1] “*even* if the entropy were proportional to the total energy, it could still fail to be proportional to the “volume” of the black hole.” Such a sentence jeopardizes the Legendre structure of classical thermodynamics, which obviously imposes that *all of its terms scale with size in exactly the same manner*. Therefore, the quantities to be legitimately compared are *U*, TS, pV, μN, HM, etc. The assumption in [1] about the possibility of the entropy being proportional to the total energy is equivalent to *a priori* assuming that *T* is intensive, a hypothesis which rather naively disregards that this issue is a very delicate one, given that, in black holes, we definitively deal with long-range interactions.) The entropic extensivity is presently verified numerically by the possible extension of the Large Deviation Theory to wide classes of strongly correlated systems [85,86,87], apparently also in [103]. The obvious mathematical convenience of using the celebrated additive entropic functional $S_{BG}$ comes from the fact that systems with local or no correlations naturally yield an extensive $S_{BG}$. In contrast, nonlocal correlations, such as those definitively existing in black holes and in many other systems, lead to a nonextensive entropy $S_{BG}$. Consequently, its use simply becomes thermodynamically inadmissible. In other words, the total entropy, the total volume, the total number of particles, the total magnetization, always belong to the same class of thermodynamical variables, sharing the property of scaling as $L^{d}$ (under the assumption that *d* is an integer number). This is in notorious contrast with the total internal and free energies which, as mentioned above, are thermodynamically extensive variables only when, say, long-range interactions are *not* involved. In this matter, it certainly is historically impressive to verify that Gibbs himself dismissed his own thermostatistical theory in those cases where the partition function diverges (e.g., gravitation) [104]. More details on the failure of BG entropy and corresponding statistical mechanics for gravitational systems can be found in [105,106,107].

## 3. Other Debatable Statements

Finally, the authors of the Comment conclude their Abstract by writing: *“We also point out other debatable statements in his analysis of black hole entropy.”*

In this context, several points can be raised, but I will restrict the focus on their statement *“we want to refer the reader to authors who have reported that (i) substituting entropy by a non-additive functional leads to inconsistent statistics [12,13,14,15] …*” (their references [12,13,14] are references [108,109,110] of the present Reply). Pessoa and Costa apparently base their conviction on the Pressé et al. interpretation of the Shore and Johnson axioms for statistical inference. It happens, however, that they are seemingly unaware that such an interpretation is deeply erroneous. Indeed, this has been discussed more than once in the literature and it has been definitively settled out by Jizba and Korbel [111], who transparently and specifically show, among others, that *$S_{q}$ does satisfy the Shore and Johnson axioms*. The authors of [1] include, as basic support of their statement about ’inconsistent statistics’, the paper [110] by Pressé et al., but no reference is made to the critical paper [112] (The title of [112] contains an unfortunate inadvertence. A more precise title would have been *Conceptual Inadequacy of the Pressé et al. Version of the Shore and Johnson Axioms for Wide Classes of Complex Systems*), where physical misconceptions and even a severe mathematical error are revealed in detail. Let us be precise about that. We straightforwardly verify $S_{q}\left(\left\{u_{i}{⊗}_{2-q}v_{j}\right\}\right)=-k\sum_{ij}\left(u_{i}{⊗}_{2-q}v_{j}\right)\ln_{2-q}\left(u_{i}{⊗}_{2-q}v_{j}\right)=-k\sum_{ij}\left(u_{i}{⊗}_{2-q}v_{j}\right)\left(\ln_{2-q}u_{i}+\ln_{2-q}v_{j}\right)\neq -k\sum_{ij}u_{i}v_{j}\left(\ln_{2-q}u_{i}+\ln_{2-q}v_{j}\right)=-k\sum_{i=1}^{W}u_{i}\ln_{2-q}u_{i}-k\sum_{j=1}^{W}v_{j}\ln_{2-q}v_{j}=S_{q}\left(\left\{u_{i}\right\}\right)+S_{q}\left(\left\{v_{j}\right\}\right)$. The crucial inequality that is present along these lines is, quite inexplicably, violated in [108].

To be more explicit, Pessoa and Costa [1] adopt the following design criteria (DC): DC1—*subdomain independence* (local information should have only local effects); DC2—*subsystem independence* (a priori independent subsystems should remain independent, unless the constraints explicitly require otherwise). As they argue, the unique functional that fits these criteria is $S_{BG}$ (and, consistently, the corresponding Kullback–Leibler relative entropy or divergence). It happens, however, that, as it becomes clear within the discussion by Jizba and Korbel in [111], DC1 and DC2 are *sufficient but not necessary* criteria for the general Shore and Johnson axioms for statistical inference. For a system with very strong space–time entanglement, such as a black-hole, hypotheses DC1–DC2 are unnecessarily restrictive.

Various other issues concerning black holes surely deserve deeper analysis, including the possibility of unification with the so-called ”area law” for strongly quantum-entangled systems, through a (conjectural) expression, such as $S_{BG}\left(L\right)\propto \frac{L^{d-1}-1}{d-1}\;\left(L\to \infty \right)$, which yields $S_{BG}\left(L\right)\propto \ln L$ for d→1 and $S_{BG}\left(L\right)\propto L^{d-1}$ for d>1 (see [113,114] and references therein). However, such challenging open problems are out of the scope of the present Reply. (Finally, a misprint appears above Equation (1) in [1], which reads ”maximization”, but should read ”minimization”.)
